# Global Burden of Alcohol Use Disorders and Alcohol Liver Disease

**DOI:** 10.3390/biomedicines7040099

**Published:** 2019-12-13

**Authors:** Jürgen Rehm, Kevin D. Shield

**Affiliations:** 1 Institute for Mental Health Policy Research, Centre for Addiction and Mental Health, 33 Russell Street, Toronto, ON M5S 2S1, Canada; kevin.david.shield@gmail.com; 2 Department of Psychiatry, University of Toronto, 250 College Street, Toronto, ON M5T 1R8, Canada; 3 Dalla Lana School of Public Health, University of Toronto, 155 College St., Toronto, ON M5T 1P8, Canada; 4 Institute of Medical Science, University of Toronto, 1 King’s College Circle, Toronto, ON M5S 1A8, Canada; 5 Campbell Family Mental Health Research Institute, Centre for Addiction and Mental Health, 33 Russell Street, Toronto, ON M5T 2S1, Canada; 6 Institute of Clinical Psychology and Psychotherapy & Center for Clinical Epidemiology and Longitudinal Studies, Technische Universität Dresden, Chemnitzer Str. 46, D-01187 Dresden, Germany; 7 Department of International Health Projects, Institute for Leadership and Health Management, I.M. Sechenov First Moscow State Medical University, Trubetskaya str., 8, b. 2, Moscow 119992, Russia

**Keywords:** alcohol, burden of disease, mortality, alcohol use disorders, alcoholic liver disease, poverty, disability-adjusted life years, interaction

## Abstract

Alcohol use is a major risk factor for burden of mortality and morbidity. Alcoholic liver disease (ALD) and alcohol use disorders (AUDs) are important disease outcomes caused by alcohol use. We will describe the global mortality and burden of disease in disability-adjusted life years for ALD and AUDs, based on data from the comparative risk assessment of the World Health Organization for 2016. AUDs have a limited impact on mortality in this assessment, since alcohol poisonings are almost the only disease category directly attributable to AUDs; most other alcohol-related deaths are indirect, and the cause which directly led to the death, such as liver cirrhosis, is the one recorded on the death certificate. Burden of disease for AUDs is thus mainly due to disability resulting from alcohol use. In contrast to AUDs, ALD is one of the major lethal outcomes of alcohol use, and burden of disease is mainly due to (premature) years of life lost. Many of the negative outcomes attributable to both AUDs and ALD are due to their interactions with other factors, most notably economic wealth. To avoid alcohol-attributable morbidity and mortality, measures should be taken to reduce the AUDs and ALD burden globally, especially among the poor.

## 1. Introduction

Alcohol use is a major risk factor for mortality and morbidity, playing a role in more than 200 diseases and injury outcomes [1,2]. However, for most regions, it can be shown that a few of the larger disease and injury categories capture more than 90% of alcohol-attributable mortality and burden of disease (the latter usually measured in disability-adjusted life-years DALYs [3]), namely alcohol use disorders (AUDs), cancer, cardiovascular disease, liver disease, and injury [4,5]. This contribution will focus on two of these major contributors to alcohol-attributable burden of disease: AUDs and alcoholic liver disease (ALD). Contrary to the other major contributors of alcohol-attributable disease, these two categories are fully alcohol attributable: there would be neither AUDs nor ALD without alcohol use [1]. Together, these two categories make up about a quarter of all alcohol-attributable mortality (26.3%) and one-third of alcohol-attributable burden of disease as measured in DALYs (31.5%; data based on [4]).

AUDs rank among the most prevalent mental disorders globally [6]. Individuals with an AUD have impaired control over their alcohol consumption and exhibit a chronic, heavy, and often escalating pattern of alcohol use despite significant detrimental consequences to their overall health, the lives of their family members and friends, and to society in general [7]; for definitions see DSM-5 [8], ICD-10 [9], or ICD-11 [10]. AUDs are very closely associated with a pattern of heavy alcohol use over time, and it has been suggested that this specific type of drinking behaviour should be part of the definition of AUDs [11,12,13].

ALD (for a definition and short overview see [14]) is the most prevalent type of chronic liver disease worldwide [15]. The International Classification of Diseases (ICD-10) recognizes several forms of ALD [14,16,17], which are usually considered as stages, that range from relatively mild and reversible alcoholic hepatic steatosis (fatty liver) and alcoholic hepatitis, to alcoholic fibrosis and sclerosis of the liver, and further to severe and irreversible stages, such as alcoholic liver cirrhosis and alcoholic hepatic failure (for a brief summary of the pathogenesis, see [18,19,20]). In some classifications, alcohol-attributable liver cancers have been listed as part of ALD as well [20,21], but we will not include this category in our calculations. Alcohol use and liver disease have an exponentially increasing dose-response relationship [22,23]. An additional impact may result from a daily heavy drinking pattern with some studies showing that the same overall amount of alcohol intake but with a non-daily drinking pattern is associated with less harm (“liver holidays” [24,25]).

AUDs and ALD are associated due to their shared etiology—alcohol use. A recent analysis of all French hospital patients between 2008 and 2012 showed that women with AUDs had 13 to 20-fold elevated risks for liver diseases (for liver cirrhosis: Hazard Ratio (HR): 19.3; 95% Confidence interval (CI): 19.1–19.5; for liver cancer: HR: 13.2; 95% CI: 12.8–13.7), and men with AUDs had 16-fold higher risks for liver cirrhosis (HR: 16.0; 95% CI: 15.9–16.1) and liver cancer (HR: 16.1; 95% CI: 15.9–16.4; [26]).

## 2. Materials and Methods

### 2.1. Definitions of Outcomes

For prevalence, we defined AUDs as being composed of alcohol dependence and harmful use of alcohol (ICD-10 [27]; or as alcohol use disorders as defined in the DSM–see above). For AUDs-attributable burden of mortality and disease, as per the usual standard of both the Global Burden of Disease study and the WHO, the following conditions were included, most notably fetal alcohol syndrome and alcohol poisoning: F10–F10.9, G31.2, G72.1, P04.3, Q86.0, R78.0, X45–X45.9, X65–X65.9, Y15–Y15.9 (GBD and WHO [4,28,29].

ALD is defined as being composed of the alcohol-attributable portion of the following ICD-10 codes: B18–B18.9, I85–I85.9, I98.2, K70–K70.9, K71.3–K71.51, K71.7, K72.1–K74.69, K74.9, K75.8–K76.0, K76.6–K76.7, K76.9 (again as per the standard definition of both the Global Burden of Disease study and the WHO [4,28,29]). The designation of “alcohol-attributable” was defined as a counterfactual to a scenario in which no alcohol was involved: in other words, all causes of death and disease with one of the above-noted ICD codes were defined as ALD if they occurred in a situation where alcohol use had been involved [30,31]. Similar definitions have been used before [15,32].

### 2.2. Data Sources

For both prevalence and attributable burden, we relied on data from the WHO Global Status Report on Alcohol and Health [4], as slightly updated by Shield and colleagues [5]. As both authors undertook the burden calculations and were authors for the Global Status Report and subsequent publications, we report not only previously published data, but also our own calculations based on the underlying data.

## 3. Results

### 3.1. Alcohol Use Disorders

Before we report on AUDs, we would like to provide some background numbers on alcohol use for the year 2016, as the prevalence of alcohol use—in particular, heavy use—and AUDs is highly correlated [4]. In 2016, more than half (57%, or 3.1 billion people) of the global population aged 15 years and older had not drunk alcohol in the previous 12 months, while some 2.3 billion people in this age group were current drinkers. Alcohol is consumed by more than half of the population in the Americas, Europe, and in the Western Pacific region, which includes China [4]. Worldwide, alcohol use has increased over the past three decades, and is expected to continue to do so, both in its prevalence and level of use [33].

**Figure 1:**
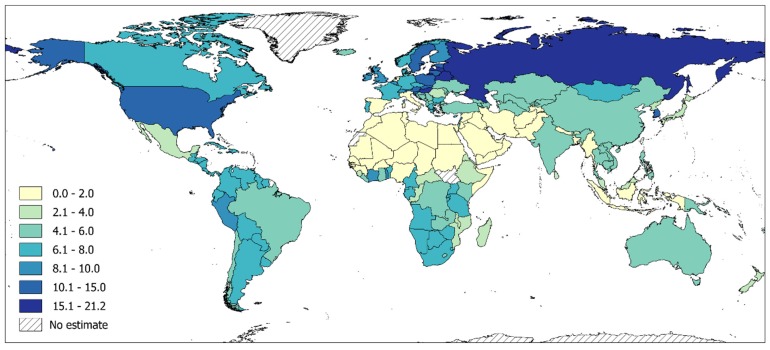
Prevalence (in %) of alcohol use disorders in adults (15 years and older) in 2016. Based on [4].

AUDs are among the most prevalent mental disorders globally, affecting 8.6% (95% CI: 8.1–9.1%) of men and 1.7% (95% CI: 1.6–1.9%) of women in 2016 (total prevalence 5.1%; 95% CI: 4.9–5.4%; see [4,6,7] for details). Although the prevalence of AUDs in men is still about five times greater than in women, in some countries—such as the US—there are signs that this gender gap is narrowing over time [34]. The prevalence of AUDs was highest in high-income countries (8.4%, 95% CI 8.0–8.9%) and upper-middle-income countries (5.4%, 95% CI 5.0–6.0%; for details on the country level, see Figure 1).

By definition, all health harms caused by AUDs were initially caused by alcohol use. In those aged 15 years or older, these harms amounted to about 145,000 AUDs deaths in 2016 (145,600; no 95% CI is available as the Global Health Estimates do not estimate CIs for cause of death or disease burden; [5,35]). A significant gender disparity exists for AUDs, due to the higher level and more detrimental patterns of drinking in men. For men, 121,600 AUDs deaths were reported while for women, a total of 24,000 was recorded.

**Figure 2:**
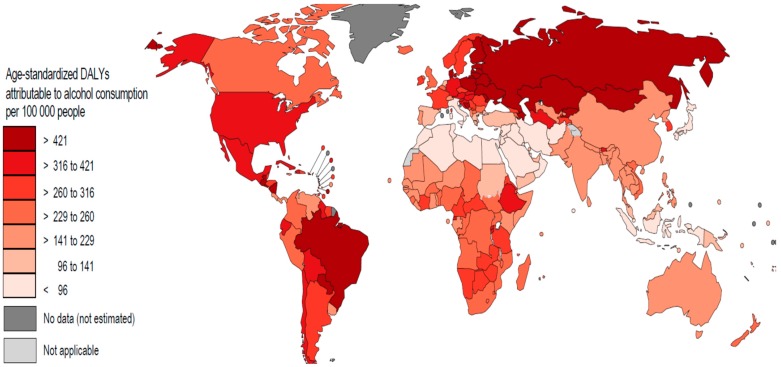
Age-standardized burden of disease (DALYs) of alcohol use disorders per 100,000 people in 2016. Based on [5].

Burden of disease figures for AUDs were proportionally higher than those for mortality. In 2016, about 21.5 million years of life were lost due to ALD (21,455,000 DALYs). Once again, men were markedly more affected than women (men: 16,614,000 DALYs; women: 4,841,000 DALYs). The majority of these years of life lost were due to disability rather than to early death. Figure 2 shows the regional distribution.

### 3.2. Alcoholic Liver Disease

Alcohol consumption caused almost half of the deaths attributed to chronic liver disease [5,36]. In the age group of 15 years and up, nearly 50% of the 1,254,000 liver disease deaths in 2016 were estimated to be alcohol-attributable (588,100; 95% CI: 531,700–683,400; 46.9% of all liver disease, 95% CI: 42.4–54.5%) [5]. This proportion is similar to previous estimates [15,32]. By gender, significantly more alcohol-attributable liver deaths were reported in men (416,700, 95% CI: 379,900–514,800) than in women (171,400, 95% CI: 134,500–189,700).

Burden of disease figures are similarly high. In 2016, about 21.5 million years of life were lost due to ALD (21,476,000 DALYs; 95% CI: 19,448,000–24,811,000); again, men were markedly more affected than women (men: 15,568,000 DALYs; 95% CI: 14,230,000–19,125,000; women: 5,909,000 DALYs; 95% CI: 4,653,000–6,423,000). The overwhelming majority of these years of life lost were due to premature deaths rather than disability.

**Figure 3:**
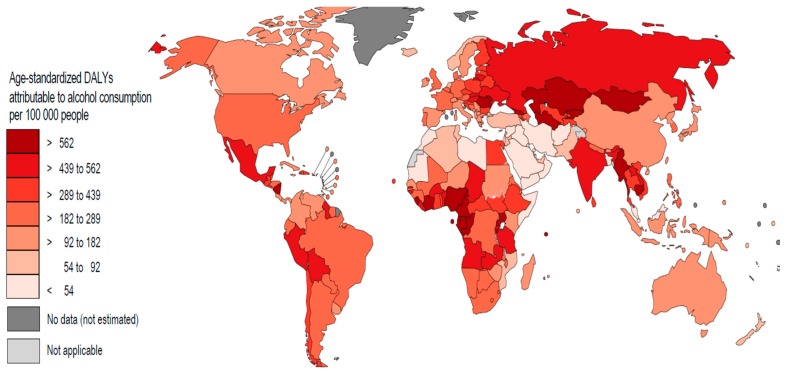
Age-standardized burden of disease (DALYs) attributable to alcohol use per 100,000 people in 2016). Based on [5].

The map in Figure 3 below shows age-standardized DALY rates per 100,000 for ALD for each WHO Member State. As expected, the lowest rates reported are seen in Southeast Asia and the Middle East, in the belt of the Muslim countries. The highest rates are found in Eastern European, West African, and Central Asian countries; the latter are relatively high in world rankings of alcohol consumption but are not necessarily among the top 20 [33].

A possible explanation for this would be that in these countries liver diseases are generally more prevalent—and are not necessarily caused by alcohol consumption, but rather by hepatitis B [37] and hepatitis C [38]. In those with existing liver disease, even a relatively small amount of alcohol can lead to serious complications or even be lethal, as shown in the significantly more exponential dose-response relationships for mortality compared to morbidity [22]. The role of alcohol use in the progression of hepatitis C was investigated in the earlier-mentioned representative cohort study of 97,347 French patients (see [26] above) with hepatitis C (the majority of the data was collected before the introduction of new methods to cure hepatitis C were available): the majority of liver complications, defined as either decompensated cirrhosis, primary liver cancer, liver transplants or liver deaths was alcohol-attributable ([39]; see also [40,41]).

Worldwide, chronic liver disease has increased significantly in overall mortality rates in the past decade (since 2010), both in terms of absolute deaths and in its ranking among major causes of death [42]. Because the alcohol-attributable share of these deaths has increased in tandem with the overall increase in chronic liver disease mortality (own calculations based on WHO, 2018), the increasing importance—from a health policy perspective—of liver disease applies especially to ALD.

## 4. Discussion

AUDs and ALD have both contributed markedly to alcohol-attributable burden of disease, with the higher contributor being ALD, especially with respect to mortality. To interpret these results, the following points and limitations should be considered:

- The definition of AUDs, and its operationalization, are both controversial, as each is based on relatively unspecific criteria (e.g., [43]), is culture-specific, and can therefore not be easily compared [44,45]. They do not necessarily coincide perfectly with heavy drinking levels and can therefore not be linked to harm [11,12] or with biological research [7,13]. While alternatives do exist to estimate AUDs [46], these are not commonly used and we therefore did not use them.
- The reported deaths resulting from AUDs only include deaths where either AUDs or alcohol poisoning was listed as the cause of death on the death certificate (see above). Similar to other mental disorders, addictive disorders such as AUDs have been associated with considerable excess mortality [47,48,49,50], even though most of these disorders do not appear in large numbers in cause-of-death statistics. The underlying cause of death in the WHO Global Health Estimates [42] is defined as the disease or injury that initiated the train of morbid events which led directly to the death, or the circumstances of the accident or violence that produced the injury. For AUDs, the cause of death would therefore be attributed to injuries such as suicides, or other chronic disorders such as ischemic heart disease or ALD [1,50,51]. However, to avoid double counting, these deaths are not counted as AUDs mortality.
- The last limitation of the estimation of AUDs prevalence and harm in this study is the reliance on the Global Health Estimates, which do not provide confidence intervals [35]. There is obviously uncertainty inherent in the estimates of AUDs and attributable harm which we could not capture.
- The burden for ALD is clearly larger than for AUDs in terms of DALYs and particularly for mortality (but see previous point). ALD is the single most important health harm caused by alcohol use [4,5]. The estimation methodology used is consistent with the definition of ALD (i.e., without alcohol use, these cases of ALD deaths or disease would not have existed). However, in contrast to other causes of death, ALD deaths were not estimated based on death certificates (in the countries where such certificates exist), or based on specific verbal autopsies [52,53], but were indirectly estimated via attributable fractions (see Materials and Methods above). The reason for this is the high stigmatization of all disorders which have the terms ‘alcohol’ or ‘alcoholic’ in their names (for general considerations, see [54]). Alcohol’s involvement in a death may be missed by those certifying or reporting the death (the latter in verbal autopsies), or may be deliberately not mentioned to protect the reputation of the deceased (see [55], for further conclusions). A landmark study of death recording practices in 12 cities in 10 countries found that the number of deaths assigned to the ICD category ‘liver cirrhosis with mention of alcoholism’ rose by 135% after taking into account additional information obtained from hospital records and interviews with attending physicians and family members. The majority of the new cases were previously coded in categories like cirrhosis without any mention of alcohol [56]. Underestimation of ALD and other 100% alcohol-attributable disease categories has persisted to this day (e.g., [57]; see short overview of a recent study in [58]).
- Finally, while there was uncertainty reported around the ALD estimates, it may be underestimated as it did not include any uncertainty for the cause of death or burden of disease (i.e., only for exposure and risk relations) [59].

Overall, AUDs and ALD have been shown to have a profound impact on the global burden of disease. Both disease categories are particularly tightly linked to heavy drinking occasions. In North America, both disease conditions have been contributing to decreasing life expectancies, especially in lower socioeconomic strata (US: [60,61,62]; Canada: [63,64,65,66]). For this region, disorders attributable to heavy alcohol consumption can, in effect, be used as an indicator for major increases in disease burden.

## 5. Conclusions

Policies to reduce the above-described burden of alcohol-attributable disease should be initiated [67]. While effective and cost-effective policies already do exist to reduce alcohol-attributable burden [67,68,69], two additional considerations should be focused on: first, each measure must be examined to ensure it does not widen the gap between socioeconomic strata and, second, the measures should target heavy drinking occasions. Thus, in addition to the raising taxes on alcohol, restricting its availability, and banning its advertisement, the current “best buys” for alcohol control—measures such as minimum pricing per unit—should be considered, as they have shown to have a strong effect on the drinking behaviour of heavy drinkers in lower socioeconomic strata [70].
